# Human chorionic plate-derived mesenchymal stem cells transplantation restores ovarian function in a chemotherapy-induced mouse model of premature ovarian failure

**DOI:** 10.1186/s13287-018-0819-z

**Published:** 2018-04-03

**Authors:** Jun Li, Qingtong Yu, Haisen Huang, Wenwen Deng, Xia Cao, Michael Adu-Frimpong, Jiangnan Yu, Ximing Xu

**Affiliations:** 1 Department of Pharmaceutics and Tissue Engineering, School of Pharmacy, Jiangsu University, Zhenjiang, Xuefu Rd, 212013 People’s Republic of China; 2 Sichuan Huahao Biotechnology Co. Ltd., Chengdu, 610041 People’s Republic of China

**Keywords:** Premature ovarian failure, CP-MSCs, Transplantation, Cyclophosphamide, Ovarian function

## Abstract

**Background:**

Previous studies have reported that transplantation of mesenchymal stem cells (MSCs) from many human tissues could ameliorate ovarian dysfunction. However, no study has revealed the therapeutic efficiency of MSCs derived from the chorionic plate (CP-MSCs) for premature ovarian failure (POF).

**Methods:**

We investigated the restorative effects of CP-MSCs on cyclophosphamide (CTX)-induced POF. The POF mouse models were established via intraperitoneal injection of 50 mg/kg CTX into female mice for 15 consecutive days. After that, CP-MSCs were intravenously transplanted into the mice once a week for 4 weeks. The serum estradiol (E2) and follicle-stimulating hormone (FSH) levels in the mouse models were detected using enzyme-linked immunosorbent assay (ELISA) before and after treatment. Ovarian function was evaluated through counting the follicles, estrous cycles, and oocytes.

**Results:**

CP-MSC transplantation restored the serum hormone level and ovarian function of the mice in the mouse model of POF induced by CTX. The levels of serum E2 and FSH in the POF model group was 232.33 ± 17.16 pg/mL and 4.48 ± 0.29 mIU/mL, respectively, after 6 weeks of treatment, which were similar to the values in the wild-type (WT) group. The superovulation demonstrated that ovarian function was significantly improved compared with nontreated POF model mice. The CP-MSC transplantation could restore CTX-induced ovarian dysfunction.

**Conclusions:**

Our results offer a potential application for human CP-MSCs in POF treatment.

## Background

Women with premature ovarian failure (POF) experience menopausal syndromes such as hot flushes, night sweats, mood swings, loss of libido, vaginal dryness, sleep disturbances, and so forth. The incidence of POF has been estimated among women prior to the age of 40 years to be approximately 1% [1]. The term premature ovarian insufficiency (POI) has also been used in addition to POF since the former reflects the spontaneous premature cessation of ovarian function which might not be “permanent”, coupled with the negative connotation implied by the term “failure” [1]. The cause of POF is idiopathic, with some cases attributed to being either spontaneous or iatrogenic. Spontaneous POF patients have abnormal ovary development due to genetic disorders, such as fragile X, Turner syndrome, and some autosomal gene mutations [2, 3]. Iatrogenic insults, such as after chemotherapy and radiation treatment for cancer, occasionally can damage ovarian function leading to premature menopause, ovarian dysfunction, and risk of infertility [1].

Currently, there is no treatment that has been approved to reverse cyclophosphamide (CTX)-induced ovarian dysfunction [4]. However, several recent discoveries have shown the potential of cell therapy for the effective improvement in ovarian function. Human bone marrow-derived mesenchymal stem cells (BM-MSCs) were the first stem cells reported to enhance ovarian function and structure in a rat model with chemotherapy-induced ovarian damage [5–7]. Subsequently, many investigators have disclosed that transplantation of mesenchymal stem cells (MSCs) from other tissues, such as amniotic fluid [8], adipose tissue [9], umbilical cord [10], menstrual blood [11], endometrium [12], and umbilical cord blood [13], could ameliorate impaired ovarian function. However, the therapeutic potential of MSCs from placental tissues in POF has not been explored.

Chorionic plate-derived MSCs (CP-MSCs) are a type of multipotent adult stem cell isolated from the chorionic plate of the human placenta, which is often treated as medical waste. Similar to BM-MSCs, CP-MSCs possess the ability for self-renewal, differentiation, homing, and secretion of growth factors [14–16]. As a new type of MSC, they have been demonstrated for the treatment of liver injury [17–20] and could be used as an alternative source of cell therapy for POF treatment. In the present study, a POF mouse model was first established via exposure to CTX, and transplantation of CP-MSCs was carried out by intravenous injection.

## Methods

### Experimental animals

Eight-week-old female specific pathogen-free (SPF)-grade C57/BL6 mice were used in our study. The mice were supplied by the Pharmacy Department, Jiangsu University (Jiangsu, China), and were fed with a standard pellet diet with free access to water. Vaginal smears were obtained daily. Only mice showing at least two consecutive normal 4- to 5-day vaginal estrus cycles were included in the experiments. The animal experimental protocol was approved by the University Ethics Committee for the Use of Experimental Animals and conformed to the Guidelines for the Care and Use of Laboratory Animals.

### Preparation and determination of CP-MSCs

All protocols were reviewed and approved by the Institutional Research Board of the Pharmacy Department, Jiangsu University, and Sichuan Huahao Biotechnology Co. Ltd. prior to the study. All stages covered in the cell preparation were processed in a current good manufacturing practice (cGMP)-compliant facility. Fresh human full-term placentas (*n* = 5) were collected after obtaining written informed consent from the patients. Placentas were stored in a sterile container at 4 °C, and the chorionic plate was separated within 24 h. Briefly, placentas were washed with saline injection three times while the chorionic plates were separated using an aseptic technique, followed by mincing into 3–5 mm^2^ explants with surgical scissors. After being washed three times, 2 g explants of chorionic plates were plated into a T-175 culture flask (Corning, USA) containing 10 mL StemPro MSC SFM (Cellgenix, Freiburg, Germany) supplemented with 1% GlutaMAX™-I CTS (Gibco, Carlsbad, CA, USA). Fresh medium was replaced every 3 to 5 days and the cell cultures were dispersed with TrypLE™ Select (Gibco) when the confluency reached 80%. The CP-MSCs were then subcultured at a density of 1 × 10^5^ cells/cm^2^ feeding with 25 mL medium, which was refreshed every 3 days.

The surface marker profiles of CP-MSCs at the third passage were tested with flow cytometry. CP-MSCs at the third passage were resuspended in cold phosphate-buffered saline (PBS) containing 2% fetal bovine serum (FBS; Gibco) at a concentration of 1 × 10^6^ cells/mL prior to addition of the following monoclonal antibodies: CD11b-FITC, CD19-ECD, CD34-PE, CD45-PC7, CD73-PE, CD90-PC5, CD105-PE, CD29-FITC, and HLA-DR-PC7 (Beckman Coulter, Brea, CA, USA). The unmarked cells were used as negative control. Finally, the stained cells were analyzed using a Beckman Coulter flow cytometry system (FC500).

Karyotype analysis of CP-MSCs at the fifth passage was performed using standard protocols for high-resolution G-banding.

Growth factors secreted by CP-MSCs at the fifth passage were also detected. Briefly, when CP-MSCs were grown to confluence, cells were refreshed with basic culture medium (DMEM; HyClone, South Logan, UT, USA) for an additional 24 h. Afterwards, the culture medium was collected and centrifuged. Enzyme-linked immunosorbent assays (ELISAs; R&D Systems, Minneapolis, MN, USA) were used to detect the levels of transforming growth factor (TGF), vascular endothelial growth factor (VEGF), fibroblast growth factor (FGF), epidermal growth factor (EGF), hepatocyte growth factor (HGF), platelet-derived growth factor (PDGF), and insulin-like growth factor (IGF)-1 in supernatant samples, with the culture medium serving as a negative control.

The multipotential differentiation of the CP-MSCs was determined for adipogenesis, osteogenesis, and chondrogenesis. Briefly, adipogenesis was induced by adipogenic differentiation medium (Cyagen, Santa Clara, CA, USA) for 21 days and confirmed by Oil Red O staining as an indicator of intracellular lipid accumulation. Osteogenesis was induced by culturing CP-MSCs in osteogenic induction medium (Cyagen) for 14 to 21 days while calcium deposition was authenticated by Alizarin red staining. CP-MSCs were also treated with chondrogenic differentiation medium (Cyagen) and chondrogenesis detected by Alcian blue staining.

### Establishment of the animal model, and stem cell transplantation

To obtain POF animal models, female C57/BL6 (*n*=80) mice were injected with CTX (50 mg/kg; Sigma-Aldrich, St. Louis, MO, USA) for 15 consecutive days. Then these POF mice were randomly divided into three groups which are POF group, CP-MSCs group and saline group. In POF group, the POF mice reveived no treatment. Mean while, in CP-MSCs group, the POF mice were transplanted with CP-MSCs (200 μL at a concentration of 2 × 10^6^ cells/kg) via the tail intravenously with a microinjector. In saline group, the POF mice were injected with 0.9% saline via tail intravenous injection. The injection was carried out once per week for 4 weeks. In addition, the wild-type (WT) group included WT mice receiving no treatment is used as blank control.

### Hormone assay

Blood samples were collected from the mice by retro-orbital puncture under anesthesia. Samples were then incubated at 20 °C for 1 h and centrifuged at 3000 × g for 10 min. The supernatant was collected to determine the levels of serum estradiol (E2) and follicle-stimulating hormone (FSH) as described in the package insert of the ELISA kits (MyBiosource, San Diego, CA, USA). Briefly, mouse E2 or FSH standards at a final concentration of 1000, 500, 250, 125, 62.5, 31.25, and 15.6 pg/mL or 20, 10, 5, 2.5, 1.25, 0.625, and 0.3125 mIU/mL or diluted mouse plasma were added to anti-E2 or FSH antibody-coated wells and incubated for 60 min. After washing three times, the horseradish peroxide (HRP)-conjugated detection antibodies were added, followed by the addition of the substrate solution. The optical density (OD) value was determined at a wavelength of 450 nm.

### Ovarian follicle counting and morphologic analysis

The mice were killed, and their ovaries were castrated at different time points following treatment. The ovaries were fixed in 4% paraformaldehyde for at least 24 h. After fixation, the ovaries were dehydrated, paraffin-embedded, serially sectioned at 5 μm and mounted on glass microscope slides. The sections were stained with hematoxylin and eosin (H&E) staining for histologic examination by light microscopy. Only follicles containing an oocyte were counted to avoid repeated counting.

### Mouse superovulation

Superovulation was carried out as previously described [9]. Mice were superovulated 2 weeks or 6 weeks after the last stem cell transplantation via an intraperitoneal injection of 5 IU pregnant mare serum gonadotropin (PMSG; Sigma-Aldrich) followed by intraperitoneal injection of 5 IU human chorionic gonadotropin (HCG; Sigma-Aldrich) 48 h later. The oocytes were collected from the ampulla portion of the oviduct 14 to 16 h after HCG injection.

### Statistical analyses

Each experiment was performed at least three times. All data were analyzed with GraphPad Prism 5 and are presented as mean ± SD. One-way analysis of variance (ANOVA) was used to determine significant differences among the four groups. In all statistical comparisons, *P* < 0.05 was taken to indicate a statistically significant difference.

## Results and discussion

### Preparation and identification of CP-MSCs

**Fig. 1:**
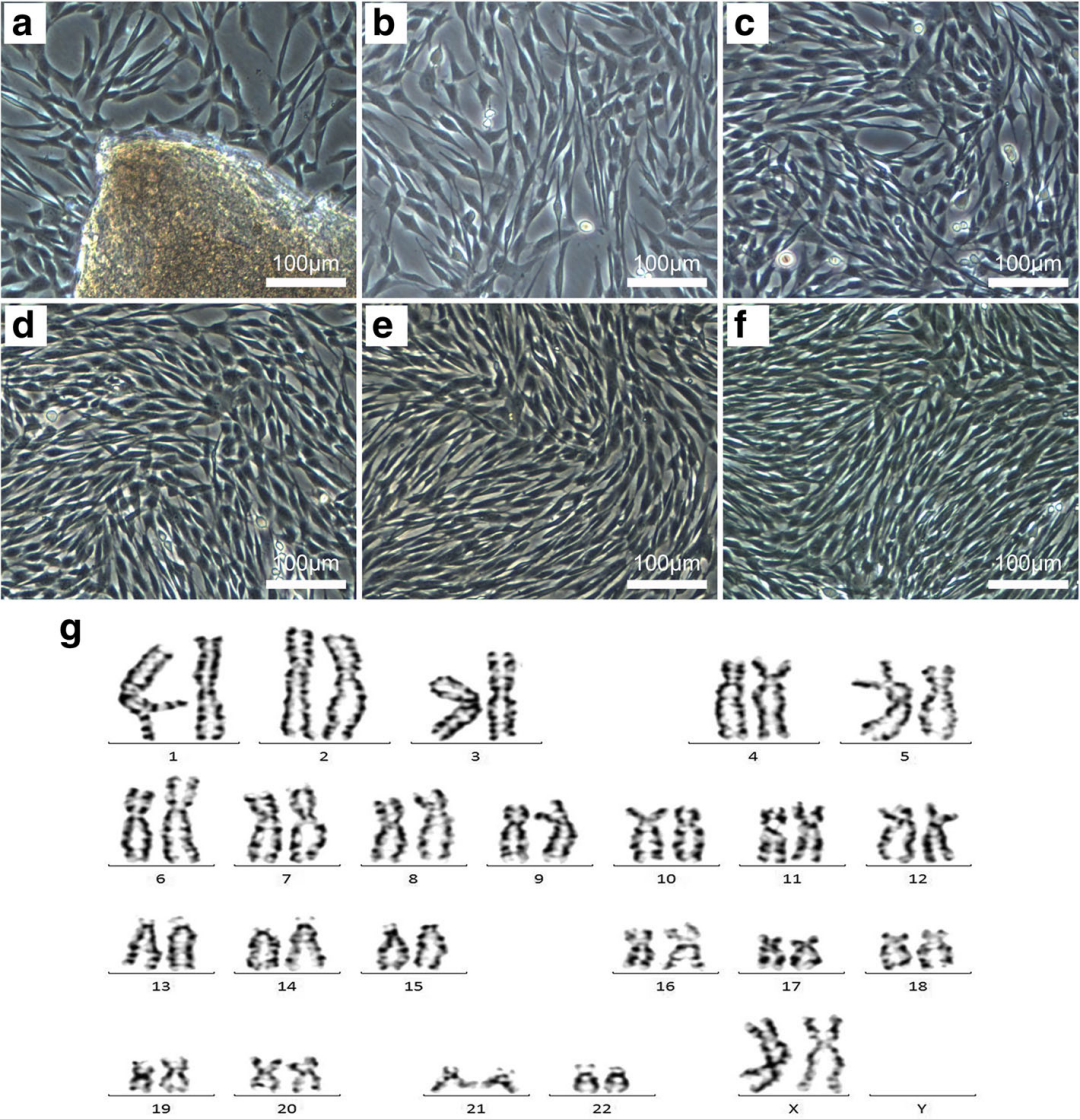
Preparation and karyotyping of CP-MSCs. CP-MSCs were isolated using the explants method (**a**) and expanded to the fifth passage (**b**–**f**, passage one to passage five). Scale bars = 100 μm. **g** Karyotyping was carried out on passage 5 CP-MSCs, and no significant chromosome variation was observed

In the present study, we designed and synthesized CP-MSCs from human placenta according to the requirements of cGMP-compliant procedures. The CP-MSCs were isolated from placental tissue via the explants method. Separated chorionic plate tissues were cultured in T-175 flasks and the primary cells were spindle-shaped and grew out of the explants after 7 to 10 days of culturing (Fig. 1a). The CP-MSCs were then serially passaged five times without any change in cell morphology (Fig. 1b–f). Since CP-MSCs at the fifth passage were used for stem cell transplantation, karyotyping was carried out to assess the safety of CP-MSCs. The results showed no significant chromosomal mutation, as indicated in Fig. 1g.

**Fig. 2:**
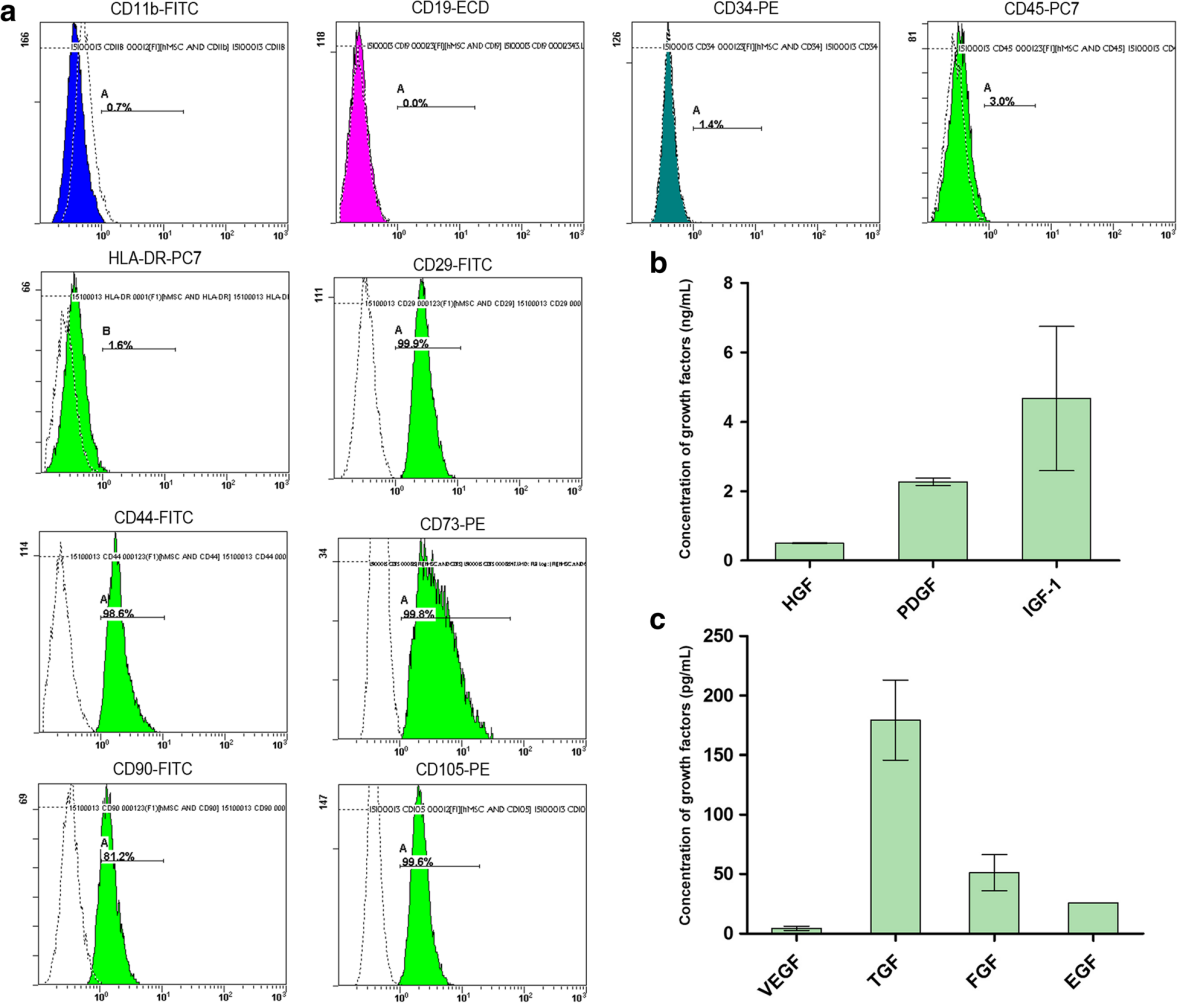
Flow cytometry and secretion of growth factors. **a** Passage 3 CP-MSCs were analyzed by flow cytometry to show the expression of surface markers CD11b, CD19, CD34, CD45, HLA-DR, CD29, CD44, CD73, CD90, and CD105. CP-MSCs were positive for CD29, CD44, CD73, CD90, and CD105, and negative for CD11b, CD19, CD34, CD45, and HLA-DR. **b**,**c** Levels of the growth factors hepatocyte growth factor (HGF), platelet derived growth factor (PDGF), insulin-like growth factor (IGF)-1, vascular endothelial growth factor (VEGF), transforming growth factor (TGF), fibroblast growth factor (FGF), and epidermal growth factor (EGF) were determined. The CP-MSCs secreted high levels of IGF-1, PDGF, and HGF, medium levels of TGF, low levels of FGF and EGF, and scarcely secreted VEGF

Flow cytometry was used to identify the expression profile of surface markers. As shown in Fig. 2a, CP-MSCs at passage 3 were negative for CD11b, CD19, CD34, CD45, and HLA-DR, and positively expressed CD29, CD73, CD90, and CD105, which are considered as surface markers for MSCs [21].

The levels of the growth factors HGF, PDGF, IGF-1, VEGF, TGF, FGF, and EGF were determined from the supernatant of spent medium collected from passage 5 culture. High levels of HGF, PDGF, and IGF-1 were detected, with levels of IGF-1 the highest at 4.67 ± 2.01 ng/mL. The concentrations of TGF, FGF, and EGF were detected at below 250 pg/mL, with TGF having the highest level (179.41 ± 33.58 pg/mL). The expression of VEGF was hardly detected Fig. 2b, c).

**Fig. 3:**
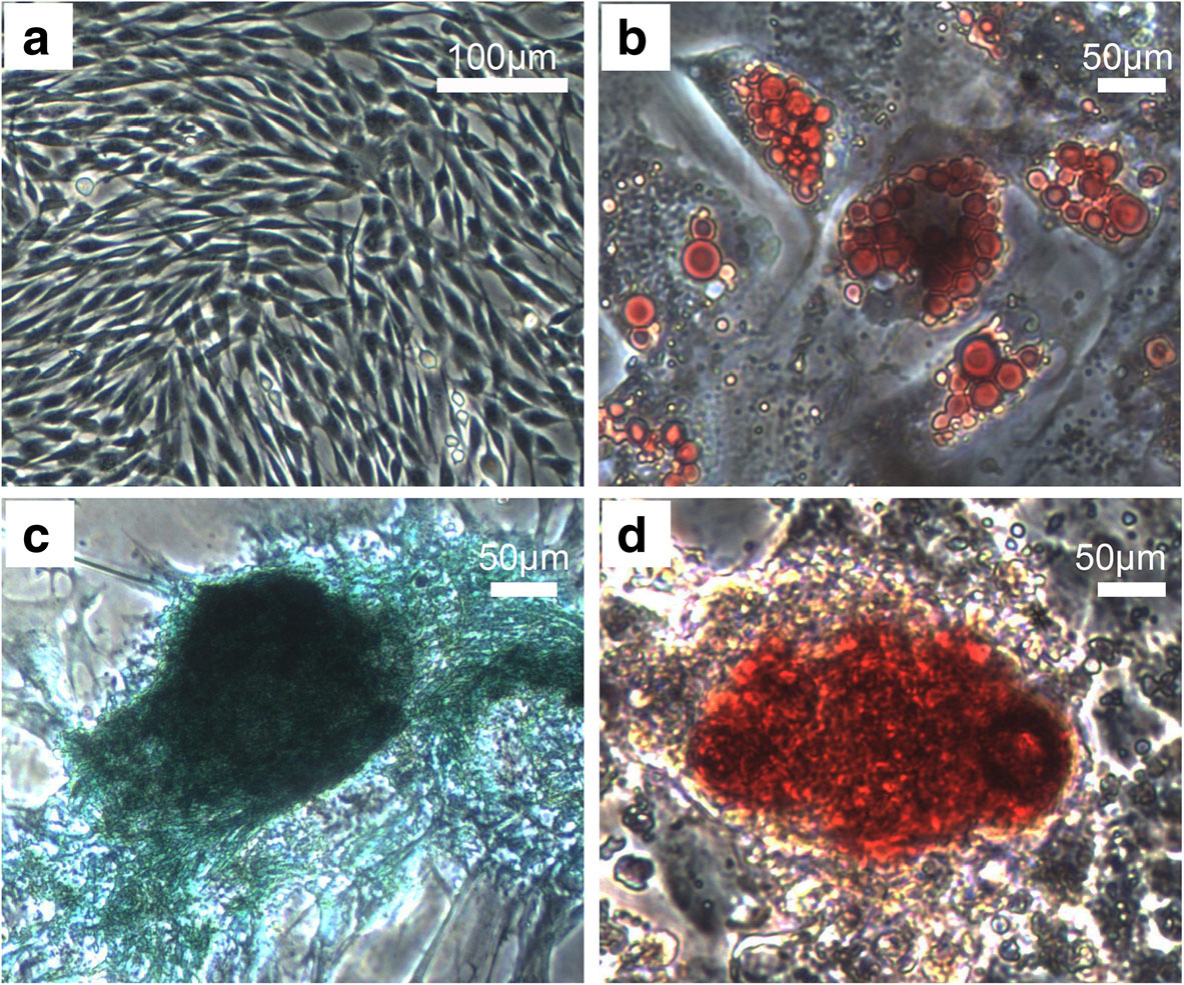
Differentiation of CP-MSCs. a Blank control; scale bar = 100 μm. CP-MSCs at passage 3 were induced to adipogenesis (**b**), chondrogenesis (**c**), and osteogenesis (**d**); scale bars = 50 μm. CP-MSCs possessed the potential for differentiation to adipocytes containing scattered lipid droplets which were stained red with Oil Red O (**b**), to chondrocytes which were stained blue with Alcian Blue (**c**), and to osteocytes with formation of calcium nodules which were stained red with Alizarin Red (**d**)

The CP-MSCs possessed multilineage differentiation as shown by their ability to orchestrate adipogenesis (Fig. 3b), chondrogenesis (Fig. 3c), and osteogenesis (Fig. 3d) when cells were cultured in the induction medium.

The data from this experiment suggest that human CP-MSCs possess characteristics of MSCs and are safe for clinical use.

### CP-MSC transplantation restores hormone levels

**Fig. 4:**
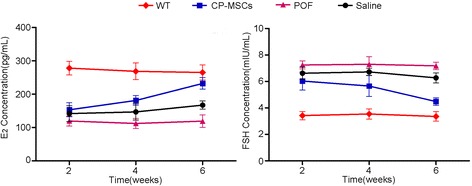
Improvement in hormone levels after placental CP-MSC transplantation. A significant improvement in the hormone level was observed in premature ovarian failure (POF) after the administration of placental chorionic plate-derived mesenchymal stem cells (CP-MSCs) (*P* < 0.05, CP-MSC treatment group versus the POF model group). The administration of saline to the POF mouse model improved the hormone level slightly; however, this change was not significant (*P* > 0.05, saline treatment group versus POF model group). The levels of estradiol (E2) and follicle-stimulating hormone (FSH) in the serum was assayed at 2, 4, and 6 weeks post-transplantation of CP-MSCs. The hormone concentration of wild-type (WT) and POF model groups were maintained at a stable level (*P* < 0.05, POF model group versus WT group). WT: wild-type mouse without any treatment as a blank control group; CP-MSCs: POF mouse model treated with CP-MSCs as an experimental group; POF: POF mouse model without any treatment as a negative control group; Saline: POF mouse model treated with saline as a vehicle group

A typical characteristic of a successfully established POF mouse model is the abnormal changes in serum levels of the female sex hormones E2 and FSH [22]. In this study, serum E2 and FSH levels were determined after CTX injection. The CTX-treated mice in the POF group had less E2 concentrations and higher FSH concentrations than the WT group. However, there was no significant change (*P* > 0.05) in hormone level for a long time even after the withdrawal of CTX, which indicates the long-term injury of CTX to ovaries of mice (Fig. 4). Four weeks after CP-MSC transplantation, the serum concentration of E2 and FSH began to partially change. The serum E2 level rose to the normal level 6 weeks after transplantation (232.33 ± 17.16 pg/mL in the CP-MSC group versus 265.00 ± 22.54 pg/mL in the WT group; *P* < 0.05). To the contrary, the serum FSH level declined from 6.03 ± 0.68 mIU/mL to 4.48 ± 0.29 mIU/mL (*P* < 0.05) as assessed against the WT group (from 3.42 ± 0.31 mIU/mL to 3.36 ± 0.36 mIU/mL; *P* > 0.05). The serum levels of E2 and FSH in the saline group did not change significantly compared with that in the POF group after treatment with saline (*P* > 0.05) (Fig. 4). These results indicated that CP-MSC cell therapy could restore the serum hormonal levels in POF mice.

### Ovarian function after treatment with CP-MSCs

**Fig. 5:**
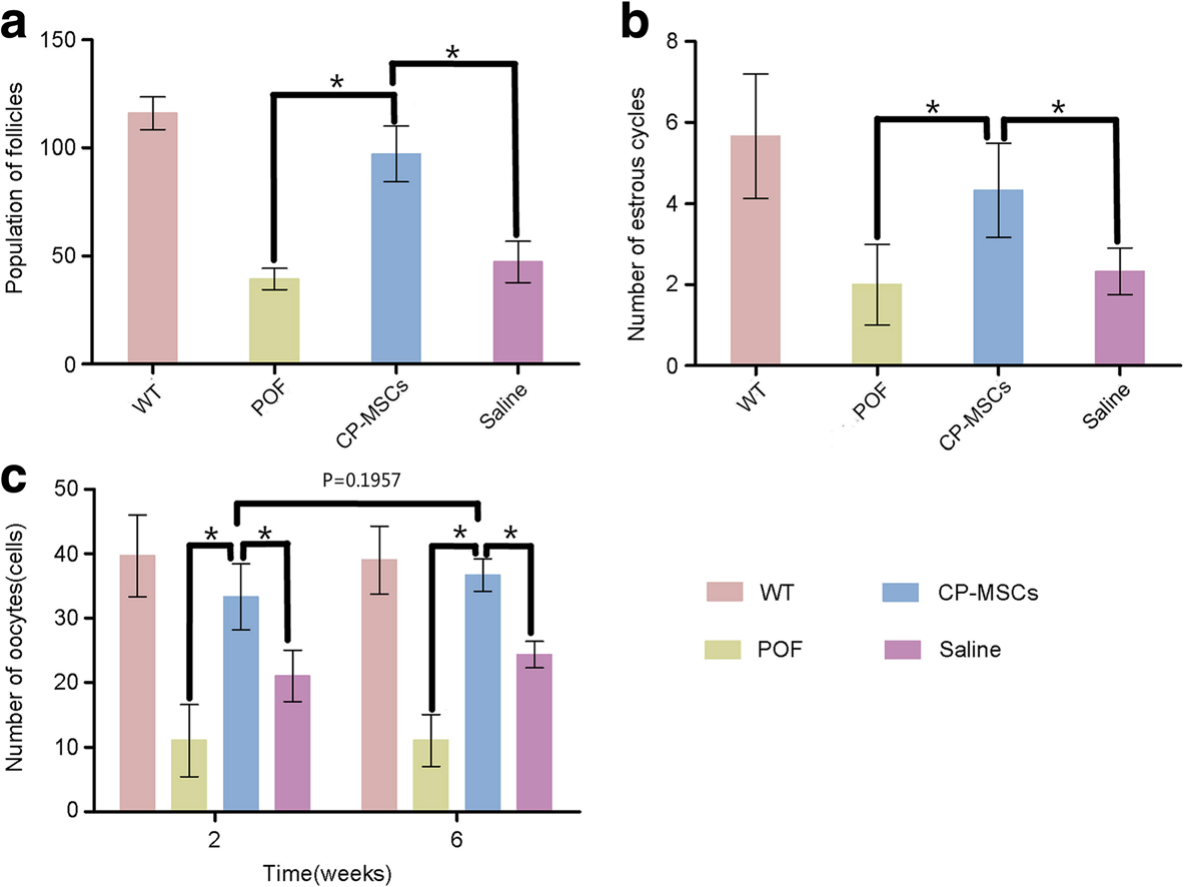
The function of the ovary in the POF mouse model improved after CP-MSC transplantation. **a** The population of follicles in the premature ovarian failure (POF) model mouse increased significantly after treatment with chorionic plate-derived mesenchymal stem cells (CP-MSCs) compared with the POF mouse model treated with saline. **b** The estrous cycles of the mice were counted post-treatment. **c** The oocytes of mice in each group were measured via superovulation to validate the ovarian function. (*P < 0.05) WT: wild-type mouse without any treatment as a blank control group; CP-MSCs: POF mouse model treated with CP-MSCs as an experimental group; POF: POF mouse model without any treatment as a negative control group; Saline: POF mouse model treated with saline as a vehicle group

Six weeks after stem cell transplantation, the follicles were counted. As shown in Fig. 5a, the population of follicles in the normal WT mouse group was 116.00 ± 7.55. The number of follicles in the POF model group was 39.33 ± 4.93 (*P* < 0.05, versus the WT group). The POF mouse model treated with CP-MSCs (CP-MSC group) increased the number of follicles to 97.33 ± 12.86 (*P* < 0.05, versus CP-MSC group) which was similar to that in the WT group. However, the POF mouse model treated with saline partly increased the follicle population (47.33 ± 9.61 follicles; *P* < 0.05 versus the CP-MSCs group).

Similar results were observed for estrous cycle counting, as shown in Fig. 5b. After cell therapy, the number of estrous cycles in the CP-MSC group rose to 4.33 ± 1.16, which was closer to that of the WT counterparts (5.67 ± 1.53 cycles; *P* < 0.05, versus the CP-MSC group). This result was much better than that observed in both the POF group (2.00 ± 1.00 cycles; *P* < 0.05) and the saline group (2.33 ± 0.58 cycles; *P* < 0.05).

Superovulation was carried out to assess whether the POF mouse model could produce oocytes after stem cell transplantation. After cell therapy, the number of oocytes in the POF group was the same at weeks 2 and 6 (11.00 ± 5.57 and 11.00 ± 4.00, respectively), whereas the number of oocytes in the WT group was 39.67 ± 6.35 at 2 weeks and 39.00 ± 5.29 at 6 weeks. Six weeks after administration of CP-MSCs, the population of oocytes ovulated in the POF mouse model increased to 36.67 ± 2.52, which was a little more than that (33.33 ± 5.13; *P* > 0.05) observed at 2 weeks after treatment. Compared with the POF group, the number of oocytes produced by the saline group slightly increased after 6 weeks of treatment to 24.33 ± 2.08 oocytes.

### Histological analysis

**Fig. 6:**
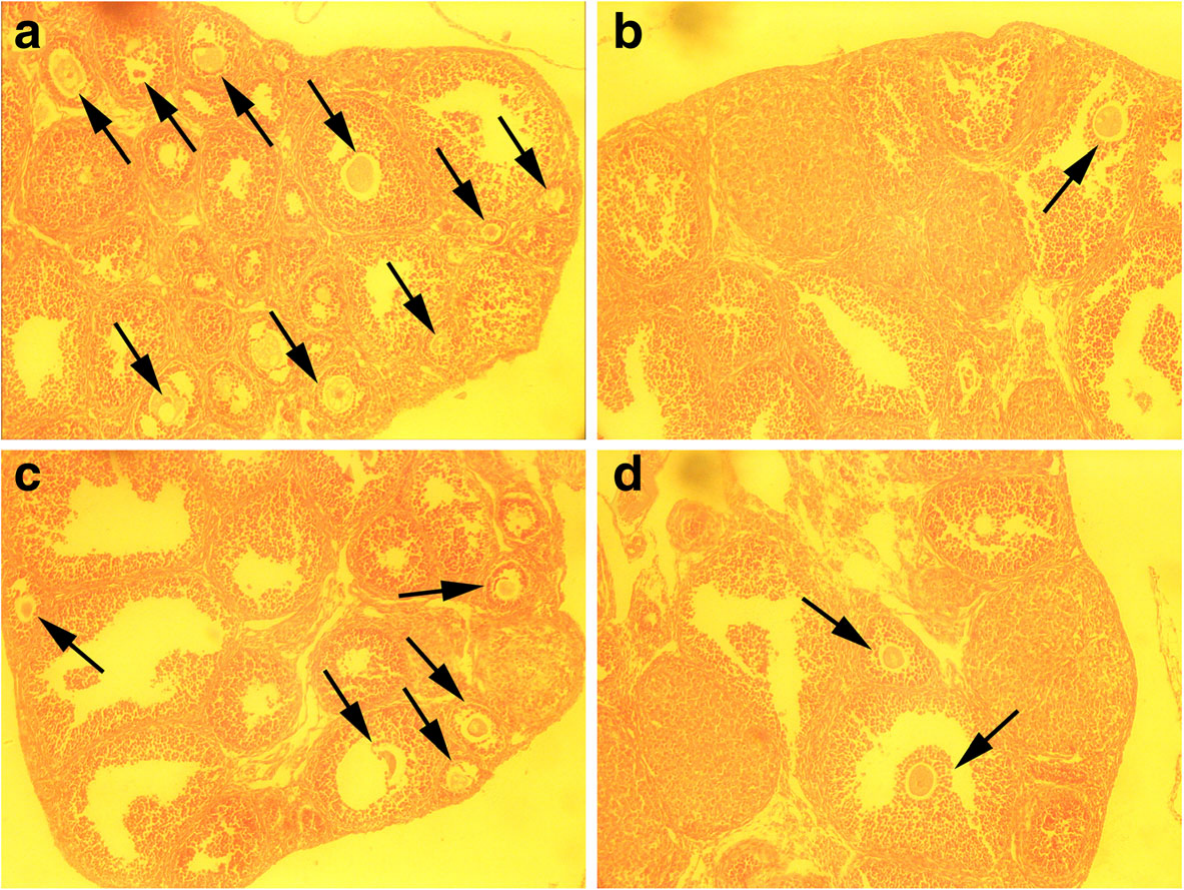
Histological analysis of mouse ovaries. Six weeks after treatment, mice ovaries were collected and fixed, followed by dehydration, paraffin embedding, and serial sectioning. The sections were stained with hematoxylin and eosin. **a** WT group, **b** POF mouse model without any treatment, **c** POF mouse model treated with CP-MSCs, and **d** POF mouse model treated with saline. Original magnification: 100×. Black arrows indicate the follicles containing a clear oocyte

Six weeks after stem cell therapy, ovaries were castrated, fixed, and sectioned. As depicted in Fig. 6b, few oocytes were observed in ovary sections derived from the POF mouse model. After treatment with CP-MSCs, the POF mouse model produced many oocytes, as indicated in Fig. 6c. To a certain degree, the ovaries of mice in the saline group were restored and produced a few oocytes (Fig. 6d).

### Discussion

The unexpected discovery of the restoration of ovarian function and fertility using bone marrow transplantation in women of reproductive age with chemotherapy-induced ovarian damage [5, 6, 23] has opened the door for the study of POF treatment using stem cells. BM-MSCs were the first stem cells used to evaluate the therapeutic potency of MSCs against chemotherapy-induced ovarian damage in a rat model [7]. In view of this, MSCs isolated from various bodily tissues have been used to recover the ovarian function damaged by chemotherapy and have shown a therapeutic prospect for POI [8–13]. Human placenta-derived MSCs (pdMSCs) were considered as good sources of MSCs for various research works in the area of regenerative medicine. Among other pdMSCs, CP-MSCs and Wharton’s jelly (WJ)-MSCs (also named umbilical cord MSCs) were considered to be more effective for cellular therapy [24]. These two cell lines share similar morphology, gene expression pattern, capacity for self-renewal, and cell surface immunophenotype. However,, CP-MSCs displayed higher expression of genes encoding the adipogenic protein adipsin and peroxisome proliferator activated receptor (PPAR)γ. Said et al. [25] studied the function of resveratrol in the ionizing radiation-induced POF model and found that the anti-inflammatory action of resveratrol might be mediated directly through suppression of NF-kB, and indirectly via enhancing PPARγ and silencing information regulator 1 (SIRT1) [25]. All the previous studies suggested that CP-MSCs might be a suitable cell source for POF therapy. In our present study, ovulation was observed 6 weeks after CP-MSC transplantation, indicating that CP-MSCs could facilitate the recovery of ovarian function. Furthermore, this observation corroborates the fact that stem cells could be used to treat various human diseases due to their self-renewal capacity and multiplex differentiation potential [8].

Hormonal imbalance with a high concentration of FSH and a low concentration of E2 in serum is a typical symptom of POF [1]. Therefore, an improvement in the female sex hormonal levels is a significant indicator of the restoration of ovarian function. In our present study, CP-MSCs were transplanted into the POF mouse model via tail vein injections, and the hormone levels were restored to normal. This finding confirms the hypothesis put forward by other investigators [9, 11].

To confirm whether CP-MSCs could facilitate improvement in ovarian function, we further studied the number of follicles and estrous cycles. After CP-MSC transplantation, the population of follicles in the CP-MSC model group rose significantly compared with that in the POF group. Similar results were observed for counting the estrous cycles. Ovulation was investigated to assess the ability of the POF mouse model to revive fertility after stem cell therapy. The number of oocytes increased compared with the control group (POF group 2 weeks after CP-MSC transplantation with the ovulation lasting at least 4 weeks).

Growth factors secreted by MSCs, such as VEGF, IGF-1, and HGF, have been suggested to play an important role in restoring ovarian function [7]. A possible mechanism is that growth factors reduce apoptosis of granulosa cells (GCs) and recover follicular development [26]. VEGF and its receptors were reported to be a critical signaling pathway in inhibiting apoptosis of GCs, and in facilitating the development of follicles [27, 28]. However, results from our current study reveal that CP-MSCs secrete low levels of VEGF and high levels of IGF-1, PDGF, and HGF. We therefore postulate that the restoration of function in chemotherapy-induced ovarian dysfunction might be through a combination of secreted growth factors, not only VEGF [27]. However, more investigations are needed to confirm the actual signaling mechanism involved in recovery of ovarian function.

## Conclusion

Our present study revealed that CP-MSC transplantation significantly restored ovarian function after CTX-induced damage. Although the underlying mechanism is still unknown, CP-MSCs could facilitate the development of follicles and oocytes. Therefore, CP-MSC transplantation might represent a promising candidate for future POF therapy, as well as for improving the quality of life in cancer survivors.

## Abbreviations

BM-MSC: Bone marrow-derived mesenchymal stem cell
cGMP: Current good manufacturing practice
CP-MSC: Chorionic plate-derived mesenchymal stem cell
CTX: Cyclophosphamide
E2: Estradiol
EGF: Epidermal growth factor
ELISA: Enzyme-linked immunosorbent assay
FGF: Fibroblast growth factor
FSH: Follicle-stimulating hormone
GC: Granulosa cell
HCG: Human chorionic gonadotropin
HGF: Hepatocyte growth factor
IGF: Insulin-like growth factor
MSC: Mesenchymal stem cell
PDGF: Platelet-derived growth factor
pd-MSC: Placenta-derived mesenchymal stem cell
POF: Premature ovarian failure
POI: Premature ovarian insufficiency
PPAR: Peroxisome proliferator activated receptor
TGF: Transforming growth factor
VEGF: Vascular endothelial growth factor
WT: Wild-type
